# Volar versus combined dorsal and volar plate fixation of complex intraarticular distal radius fractures with small dorsoulnar fragment – a biomechanical study

**DOI:** 10.1186/s12891-021-04989-w

**Published:** 2022-01-05

**Authors:** Mariya Hadzhinikolova, Ivan Zderic, Daniel Ciric, Jan P. Barcik, Dian Enchev, Asen Baltov, Lyubomir Rusimov, Peter Varga, Karl Stoffel, Geoff Richards, Boyko Gueorguiev, Mihail Rashkov

**Affiliations:** 1 AO Research Institute Davos, Davos, Switzerland; 2 Department of Trauma Surgery, University Multiprofile Hospital for Active Treatment and Emergency Medicine ‘N. I. Pirogov’, Sofia, Bulgaria; 3 Flinders University, Tonsley, South Australia; 4 University Hospital Basel, Basel, Switzerland

**Keywords:** Complex intraarticular distal radius fracture, Volar plate, Double plating, Dorsoulnar fragment fixation, Biomechanical testing

## Abstract

Complex intraarticular distal radius fractures (DRFs), commonly managed with volar locking plates, are challenging. Combined volar and dorsal plating is frequently applied for treatment, however, biomechanical investigations are scant. The aim of this biomechanical study was to investigate volar plating versus double plating in DRFs with different degrees of lunate facet comminution.

Thirty artificial radii with simulated AO/OTA 23-C2.1 and C3.1 DRFs, including dorsal defect and lunate facet comminution, were assigned to 3 groups: Group 1 with two equally-sized lunate facet fragments; Group 2 with small dorsal and large volar fragment; Group 3 with three equally-sized fragments. The specimens underwent volar and double locked plating and non-destructive ramped loading in 0° neutral position, 40° flexion and 40° extension.

In each tested position, stiffness: (1) did not significantly differ among groups with same fixation method (*p* ≥ 0.15); (2) increased significantly after supplemental dorsal plating in Group 2 and Group 3 (*p* ≤ 0.02).

Interfragmentary displacements between styloid process and lunate facet in neutral position were below 0.5 mm, being not significantly different among groups and plating techniques (*p* ≥ 0.63).

Following volar plating, angular displacement of the lunate facet to radius shaft was significantly lower in Group 1 versus both Group 2 and Group 3 (*p* < 0.01). It decreased significantly after supplemental dorsal plating in Group 2 and Group 3 (*p* < 0.01), but not in Group 1 (*p* ≥ 0.13), and did not differ significantly among the three groups after double plating (*p* ≥ 0.74).

Comminution of the lunate facet within its dorsal third significantly affected the biomechanical outcomes related to complex intraarticular DRFs treated with volar and double locked plates.

Double plating demonstrates superior stability versus volar plating only for lunate facet comminution within its dorsal third. In contrast, volar plating could achieve stability comparable with double plating when the dorsal third of the lunate facet is not separated by the fracture pattern. Both fixation methods indicated achievable absolute stability between the articular fragments.

## Introduction

**Fig. 1:**
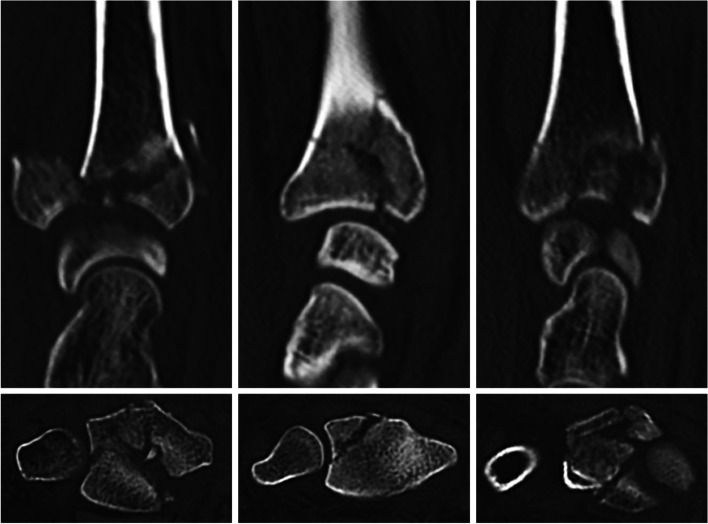
Coronal (top) and axial (bottom) computed tomography scans of clinical cases with intraarticular distal radius fractures with different degree of lunate facet comminution comprising two fragments of a comparable size (left), a smaller dorsal and a larger volar fragment (middle), and three fragments of a comparable size (right)

Volar locking plates have established a standard for reliable fixation across the wide spectrum of distal radius fractures (DRFs) [1]. Approximately 80% of the intraarticular DRFs can be treated with a single volar plate [2]. A subset of DRFs present a typical comminution pattern of the lunate facet comprising a volar ulnar corner rim fragment, a dorsoulnar fragment, and possibly a free or impacted intraarticular fragment (Fig. 1).

This intermediate column fragmentation, observed in both osteoporotic and healthy bone, is recognized as challenging and prone to complication [3–5]. Non-anatomical reduction of the ulnovolar rim fragment has been reported to render the carpal joint susceptible to volar subluxation [6]. On the other hand, the dorsoulnar fragment often comprises part of both the radiolunate and radioulnar articular surfaces and is crucial in maintaining appropriate sagittal radiocarpal alignment and preventing dorsal collapse [7, 8].

Fixed-angle volar locking plates provide reliable fracture fixation in case of osteoporotic and/or comminuted fractures [9]. This is further facilitated by the variable-angle locking plate technology allowing better targeting and purchase in the densest available subchondral bone. However, in the case of extremely comminuted and unstable fractures, a single volar plate may not provide sufficient stability to the dorsal rim, thus necessitating supplemental dorsal fixation. Combined volar and dorsal plate fixation of intraarticular fractures with comminution of both the metaphysis and the articular surface has been clinically studied [10]. However, in the extensive body of literature on the biomechanical and clinical performance of volar locking plates, there is a paucity of evidence as to the indications of dorsal plate augmentation in the setting of volar plate fixation. Moreover, no studies have subjected these two fixation methods to a direct comparison in order to provide reliable data on the biomechanical behaviour of fracture models with different degrees of comminution.

Therefore, the aim of this study was to investigate the biomechanical competency of volar-plated DRFs compared with double plate fixation in three complex fracture models with different degrees of lunate facet comminution.

We tested the hypothesis that a supplemental dorsal plate would be needed to preserve the integrity of fractures separating the dorsal third of the lunate facet.

## Materials and methods

### Specimens and preparation

Thirty artificial right radii (#7001, cortical bone density 0.79 g/cm^3^, cancellous bone density 0.17 g/cm^3^, SYNBONE AG, Zizers, Switzerland) were assigned to three treatment groups with ten specimens each, simulating plated complex three- or four-part intraarticular AO/OTA 23-C2.1 or C3.1 fractures with different degrees of lunate facet comminution.

**Fig. 2:**
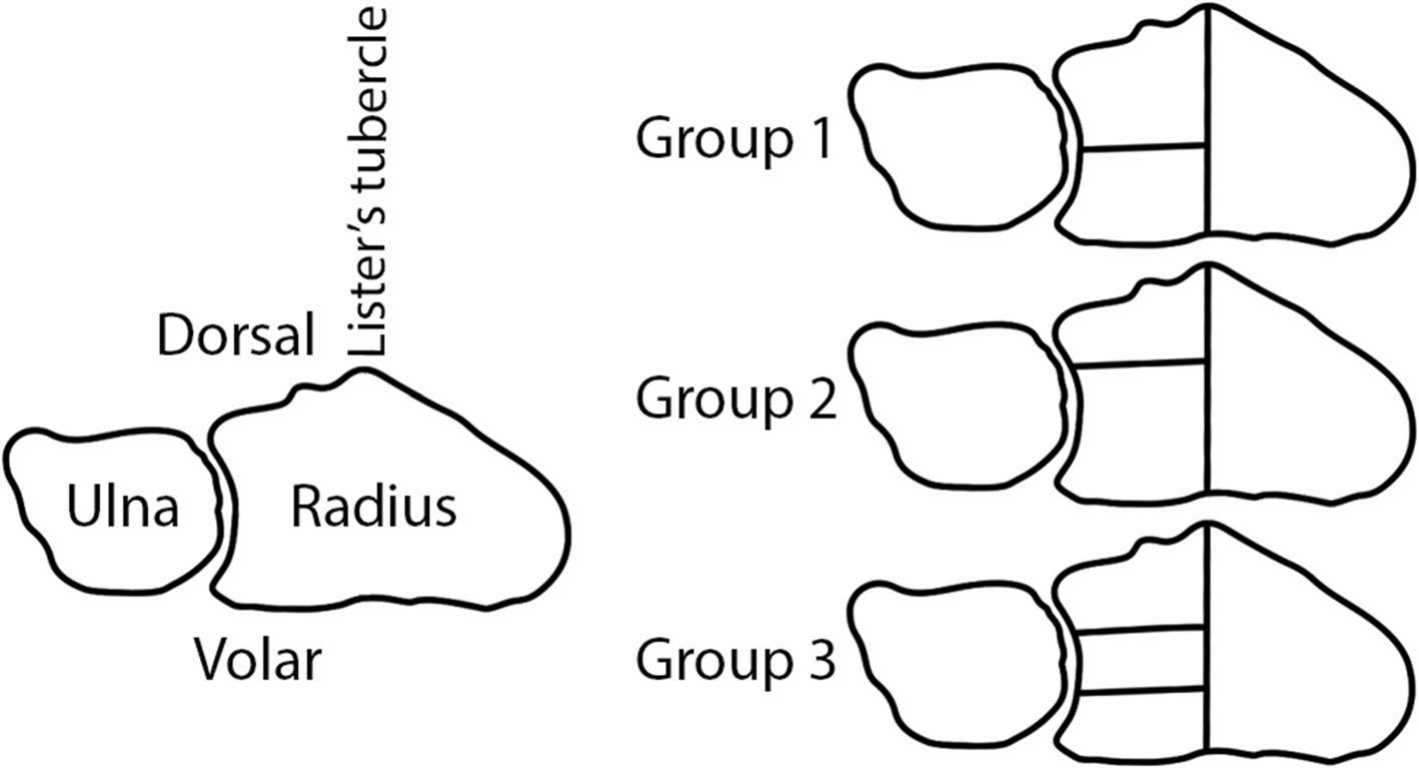
Schematic illustration of the distal radius and ulna in axial view (left), together with the group assignment according to the intraarticular fracture patterns at the lunate facet (right) with two equally-sized fragments (Group 1, top right), with a small dorsal and a large volar fragment at a ratio of 1:2 (Group 2, middle right), and with three equally-sized fragments (Group 3, bottom right)

The fracture models were created as follows. First, a 15° dorsal wedge-shaped osteotomy gap, located 10 mm proximal to the articular surface to mimic comminution of the dorsal distal radius aspect, was created in all specimens. Second, an intraarticular fracture line was set through the Lister’s tubercle in the sagittal plane. The styloid process of the radius was then separated from the shaft and the remaining articular fragments as a single piece. Third, the lunate facet was split by coronal osteotomies, so that it consisted of two equally-sized fragments in Group 1**,** a small dorsal and large volar fragment at a ratio of 1:2 in Group 2, and three equally sized fragments in Group 3 (Fig. 2).

**Fig. 3:**
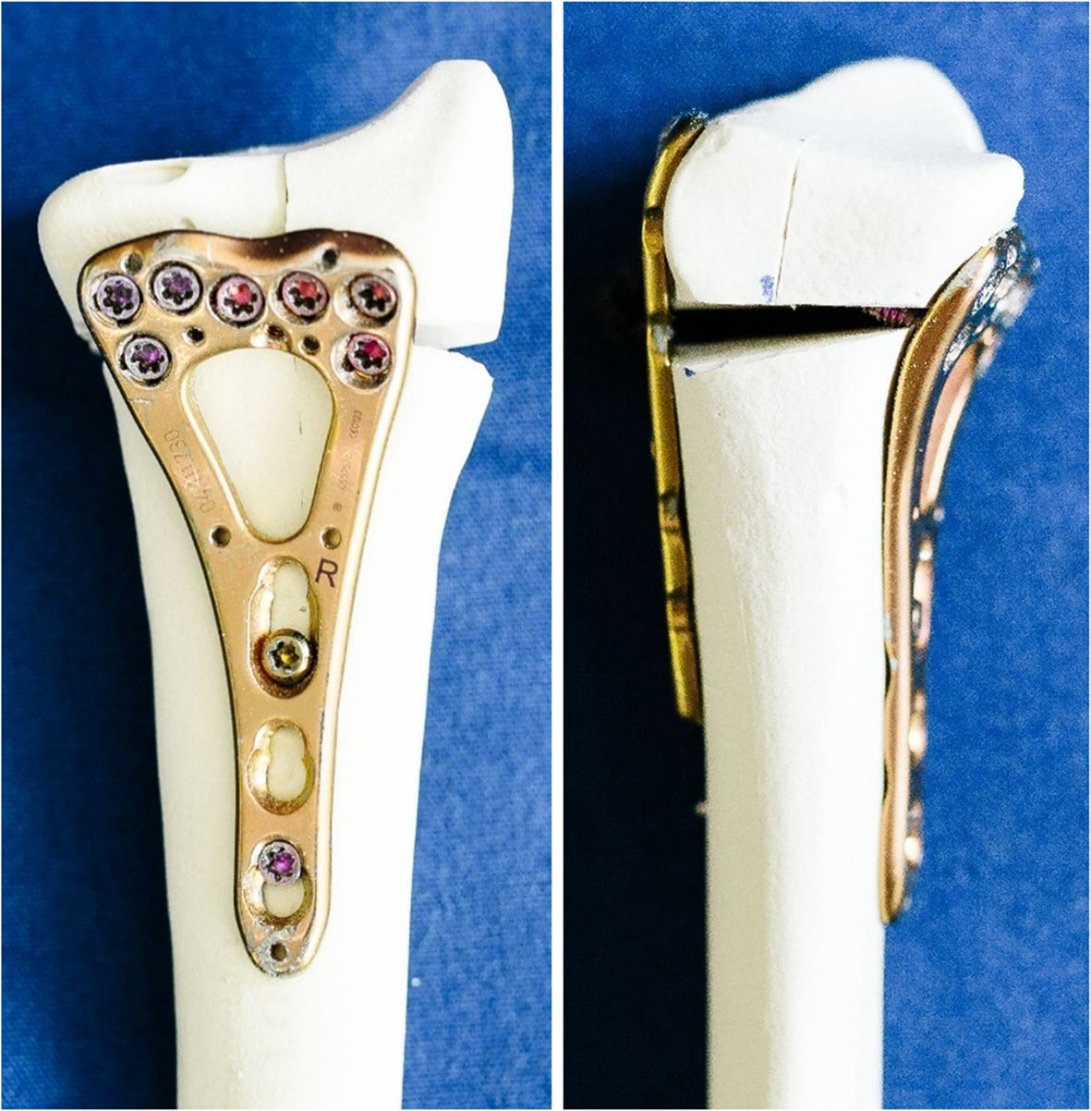
Exemplified specimens from Group 2 after volar plating using a 2.4 mm Variable Angle LCP Two-Column Volar Distal Radius Plate (DePuy Synthes, Zuchwil, Switzerland) (left), and double plating using a supplemental Variable Angle LCP Dorsal Distal Radius Plate (DePuy Synthes, Zuchwil, Switzerland) (right)

Following fracture reduction, all radii were first double-plated using a 2.4 mm Variable Angle LCP Two-Column Volar Distal Radius Plate (DePuy Synthes, Zuchwil, Switzerland) and a 2.4 mm Variable Angle LCP Dorsal Distal Radius Plate (DePuy Synthes, Zuchwil, Switzerland) – made of Ti-6Al-7Nb alloy – according to the manufacturer’s recommendations (Fig. 3).

All locking screws were inserted according to the corresponding surgical guidelines, applying widely accepted techniques to avoid dorsal compartment penetration of the volar screws [11–13]. The insertion of each screw was performed at 90° with respect to its hole axes, except for the middle screw in the proximal volar plate row, which was directed to the lunate facet fragment with an angulation of 15°. The diaphyseal screws of the volar plate were bicortical, whereas its articular screws were monocortical with lengths in the range 18–22 mm (depending on the specimen’s anatomy) and inserted subchondrally at a distance of 2 mm from the far cortex. All screws of the dorsal plate were monocortical with a length of 22 mm. The specimens were cut proximally to a total length of 170 mm. Two Kirschner (K-) wires were inserted into the radial shaft and one of the lunate fragments, oriented in the sagittal plane for radiological evaluation in mediolateral (ML) view. Additionally, metal balls were inserted in the volar and dorsal fragments of the lunate facet, and in the styloid process to assess their interfragmentary displacements via radiological evaluation in anteroposterior (AP) view.

### Biomechanical testing

**Fig. 4:**
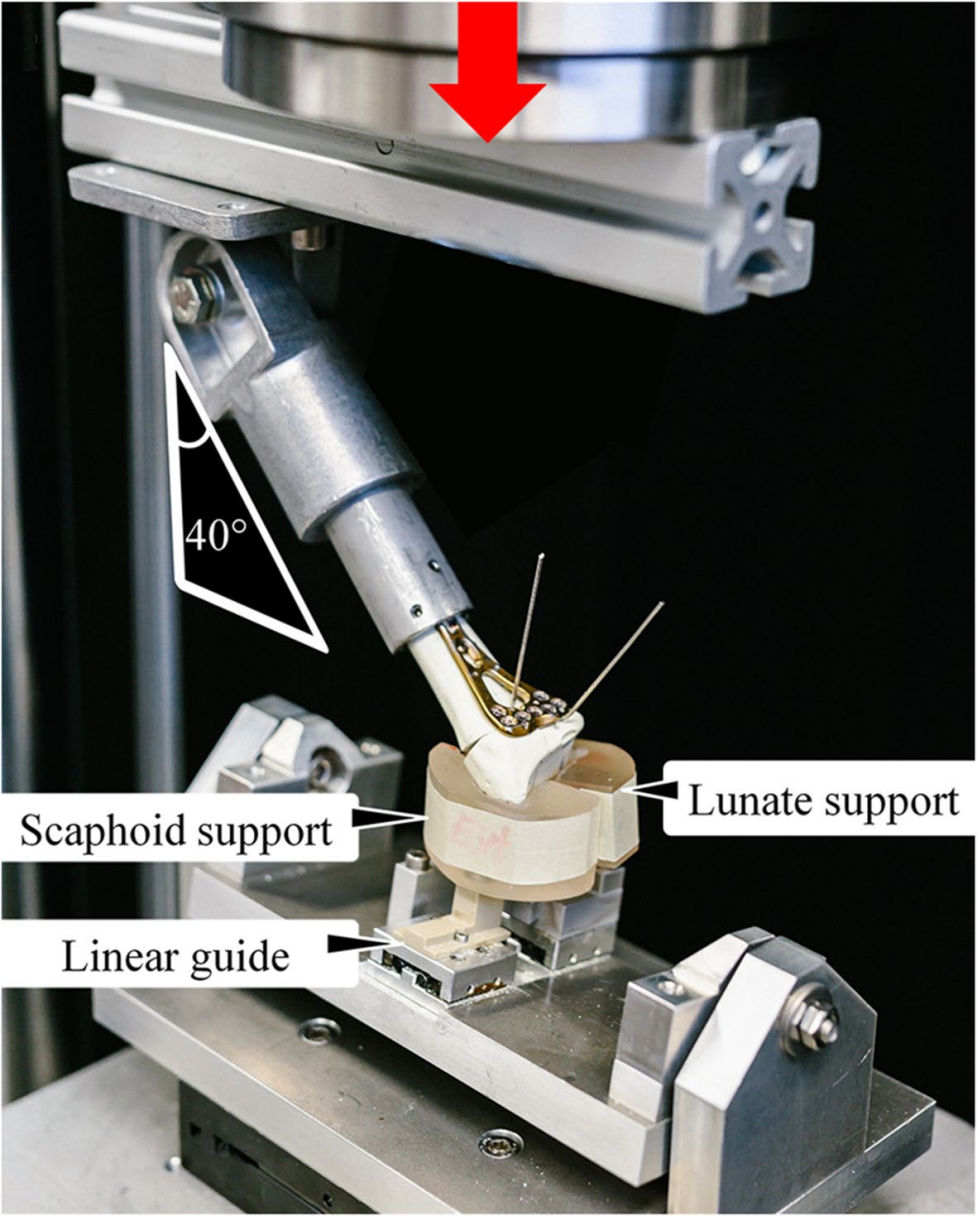
Test setup with a specimen mounted for biomechanical testing in extension, with vertical arrow denoting the loading direction, and indicated inclination angle of the radial shaft

Biomechanical testing was performed on a servo-hydraulic material testing system (Mini Bionix II 858, MTS Systems Corp., Eden Prairie, MN, USA) equipped with a 4 kN loadcell. Each specimen was non-destructively loaded along the machine axis using three setups featuring neutral position along the shaft axis, flexion or extension of the radius (Fig. 4). For this purpose, the proximal end of each specimen was coupled to the machine actuator via a custom-made fixation with a set angle of 90° for neutral position and 40° for flexion and extension. The distal load transfer to the articular surfaces was achieved via two custom-molded polymethylmethacrylate (PMMA, SCS-Beracryl D28, Suter Kunststoffe AG, Fraubrunnen, Switzerland) supports, one supporting the scaphoid facet, and the other the lunate facet. Three different separate sets of supports were negatively casted for testing in neutral position, flexion and extension. Both supports rested on a metal sphere of 8 mm diameter, enabling all rotational degrees of freedom. The spheres were positioned asymmetrically on a custom-made seesaw to achieve a predefined load distribution of 60% and 40% transmitted to the scaphoid and lunate facets, respectively [14, 15]. In addition, the sphere under the scaphoid facet was supported on a miniature linear guide for free mediolateral movement. Finally, the seesaw was mounted on the machine base with an XY table to alleviate shear forces.

The protocol for all tests consisted of a non-destructive ramped loading from 10 N to 100 N at a rate of 10 N/sec. The linear elastic behavior of the plated specimens under these loading conditions was confirmed in pilot tests. The upper limit of 100 N was defined based on previous studies [16–19]. From a clinical perspective, it was within the expected range of physiological loading for light wrist motion. The loadcell operated with a measurement uncertainty of 0.4% within a load range 0–250 N and was therefore suitable for the applied loads.

Following the biomechanical testing of the double-plated specimens, their dorsal plate was removed and all tests were repeated using a single volar plate fixation.

### Data acquisition and analysis

Force and crosshead displacement machine data were acquired at 128 Hz. Based on these, construct stiffness of each specimen was calculated from the linear elastic region of the force-displacement curve within a load range 40–80 N for each loading direction and type of plate fixation. Moreover, all tests were accompanied by ML and AP radiographs taken at 10 N and 100 N force levels with a C-arm (ARCADIS Varic VC10A, Siemens Healthineers, Erlangen, Germany). Based on the ML radiographs, angular displacement of the lunate facet fragments was measured with respect to the radius shaft in the sagittal plane by evaluating the angle change between the K-wires. Interfragmentary displacements between the styloid process and the lunate facet in the coronal plane were measured from the AP radiographs by the change in distance between the metal spheres in neutral specimen’s position.

Statistical analysis was performed using SPSS software package (v.27, IBM SPSS, Armonk, NY, USA). Normal distribution of the data was screened and proved with Shapiro-Wilk test. One-Way Analysis of Variance (ANOVA) with Bonferroni Post-Hoc test for multiple comparisons and Paired-Samples t-test were applied to identify significant differences between the study groups, fixation methods and radius inclinations during testing. Level of significance was set to 0.05 for all statistical tests.

## Results

The results for construct stiffness, angular displacement and interfragmentary displacement are summarized in Table 1.

**Table 1:** Stiffness (ST), angular displacement (AD) and interfragmentary displacement (ID) in the three study groups (GR) for volar (V) and double (D) plating (PL) under neutral (N) loading condition, flexion (F) and extension (E), in terms of mean and standard deviation. *P*-value (P) indicates statistical differences between volar and double plating for the respective outcome. Bold values indicate significant differences

GR	PL	ST [N/mm]			AD [°]			ID [mm]
		N	F	E	N	F	E	N
**1**	**V**	187.5 (48.4)	51.4 (14.2)	41.5 (11.3)	0.38 (0.18)	0.75 (0.26)	0.88 (0.32)	0.19 (0.07)
	**D**	197.5 (51.6)	55.6 (15.9)	48.9 (15.4)	0.28 (0.10)	0.67 (0.19)	0.72 (0.24)	0.17 (0.06)
	***P***	*0.12*	*0.15*	*0.27*	*0.13*	*0.27*	*0.14*	*0.81*
**2**	**V**	158.5 (44.4)	41.5 (13.7)	35.7 (9.9)	1.23 0.35)	1.95 (0.53)	2.42 (0.75)	0.22 (0.10)
	**D**	196.5 (50.5)	54.4 (15.3)	47.5 (14.9)	0.31 (0.13)	0.71 (0.22)	0.76 (0.27)	0.20 (0.08)
	***P***	***< 0.01***	***< 0.01***	***0.02***	***< 0.01***	***< 0.01***	***< 0.01***	*0.41*
**3**	**V**	155.9 (44.2)	40.7 (13.1)	34.7 (9.2)	1.32 (0.47)	2.11 (0.62)	2.66 (0.79)	0.23 (0.10)
	**D**	189.3 (49.7)	53.8 (14.9)	46.9 (13.5)	0.34 (0.15)	0.74 (0.23)	0.82 (0.29)	0.22 (0.09)
	***P***	***< 0.01***	***< 0.01***	***< 0.01***	***< 0.01***	***< 0.01***	***< 0.01***	*0.75*

In neutral position, flexion or extension, construct stiffness remained without significant differences between the three study groups treated with same plate fixation – volar or double plating (*p* ≥ 0.15). However, for each separate group and fixation method, stiffness in neutral position was significantly higher versus both flexion and extension (*p* < 0.01), being not significantly different between flexion and extension (*p* ≥ 0.13). In each tested position, stiffness increased significantly after supplemental dorsal plating in both Group 2 and Group 3 (*p* ≤ 0.02), but not in Group 1 (*p* ≥ 0.12).

Following volar plating, angular displacement in neutral position, flexion or extension was significantly lower in Group 1 compared with both Group 2 and Group 3 (*p* < 0.01), being not significantly different between Group 2 and Group 3 (*p* ≥ 0.93). In each tested position, angular displacement decreased significantly after supplemental dorsal plating in both Group 2 and Group 3 (*p* < 0.01), but not in Group 1 (*p* ≥ 0.13), and did not demonstrate any significant differences between the three groups after double plate fixation (*p* ≥ 0.74). In addition, for each separate group and fixation method, angular displacement in neutral position was significantly lower versus both flexion and extension (*p* ≤ 0.03), whereas it did not differ significantly between the latter two inclinations (*p* ≥ 0.14).

Interfragmentary displacements between the styloid process and lunate facet in neutral position were below 0.5 mm and did not reveal any significant differences among the study groups and fixation methods (*p* ≥ 0.63).

## Discussion

This study compares the biomechanical competency between volar locked plating and combined dorsal and volar double plate fixation of complex DRFs. We used three fracture models with different degrees of comminution to investigate the impact of lunate facet separation on the fracture stability.

Our investigation proves that when the dorsal third of the lunate facet is not separated by a fracture line, volar locked plating and double plating achieve comparable stability.

In contrast, double-plated constructs demonstrated superior fixation stability compared with a single volar locked plating in cases of lunate facet comminution within its dorsal third.

Currently, no existing studies seem to conclude and recommend if and when additional dorsal support would be necessary for reliable fixation of fracture patterns featuring the various specific types of lunate facet fragmentation.

Several biomechanical studies have focused primarily on comparisons between various volar or dorsal plate fixations [20–24]. However, a relationship between the stability of those constructs and the degree of lunate facet comminution has not been investigated.

We considered it justified to contrast the existing reports – advocating single volar locked plating as being able to provide reliable stability for most dorsally comminuted DRFs – via a carefully designed biomechanical experiment in the current study, in order to provide evidence answering the question which cases would require additional dorsal support [24–28].

In our study, Group 1 implemented a reference fracture model based on previous biomechanical work [15, 25, 26]. In accordance with previous investigations, three different test setups and protocols with non-destructive axial compression and volar or dorsal bending of the distal radius were developed to resemble early postoperative wrist motion and grasp [25–31].

In the current clinical practice, most dorsally comminuted DRFs are treated with volar instead of dorsal locking plates – an algorithm followed with insufficient evidence [8, 32, 33].

A dorsal approach has been suggested for some intraarticular fractures [1, 25], especially with lunate facet comminution involving dorsoulnar fragmentation. Dorsal plate application is performed with an additional surgical approach, which can result in additional surgical trauma and higher complication rates due to the small volume of the dorsal compartments. Most common complications are extensor tendon ruptures and tenosynovitis [12, 15, 34–43].

The need for dorsal support and several findings of our study can be explained with the mechanism of loading and injury of the lunate facet. The three-column theory of the distal radius was introduced in the context of dorsal buttress double plating and later corroborated in several in-vivo and in-vitro studies [30, 44]. The fragmentation of the lunate facet is a direct consequence of the loading conditions described by this theory. Secure fixation of the intermediate column – heavily loaded in compression – has attracted the interest of a number of investigators [15, 22, 23, 45–48].

A previous investigation reported on the bending moments acting on the bone-implant constructs and concluded that they increase as the point of the respective force application moves away from the plate [43]. Therefore, the more dorsally the point of force application is located, the greater the mechanical disadvantage will become, which can be counteracted with a volar plate.

The load transmission at the distal radius was shown to be concentrated on the volar side during flexion and more dorsally during extension [49, 50]. In contrast, a previous investigation, comparing in-vitro and in-vivo results, concluded that the load distribution to the lunate facet in flexion and extension is generally equalized [30]. In our test setups we implemented different distal load distributions for flexion and extension of the distal radius but the results were still comparable between these two positions for each separate fracture model and fixation method.

A number of clinical studies demonstrated that small volar DRF fragments are challenging to address and that an unstable fixation may result in radiocarpal and radioulnar joint subluxations [4, 5, 28, 51].

The importance of the dorsoulnar fragment and its contribution to construct stability has attracted rather little attention and the few available reports provide conflicting evidence. It is known that this fragment of the distal radius articular surface contributes considerably to the congruency of the distal radioulnar joint [7, 52]. Moreover, it plays a crucial role in the maintenance of an adequate sagittal radiocarpal alignment and the prevention of dorsal collapse [2].

In several cadaveric biomechanical studies, the change in contact pressure of the wrist was investigated for step-off intraarticular malunions [53, 54]. A step-off of 1 mm or more within the volar lunate facet was found to increase the contact pressure in the radiocarpal joint, whereas no considerable contact pressure change was reported at an articular step-off of up to 2 mm within the dorsal distal radius [55]. These findings could explain why a volar lunate facet incongruity leads to poor outcomes, whereas a displaced dorsal rim fragment does not [53].

A finite element investigation reported that in presence of a 1 mm step-off within the volar lunate facet, the contact stress distributions shifted towards the ulna [55]. A retrospective study demonstrated that the size of the dorsoulnar fragment is not associated with occurrence of postoperative fracture displacement [2]. Although fixation of this fragment – shared by the distal radioulnar and radiocarpal joints – appears valuable in preventing postoperative fracture dislocation, there is no existing consensus on its optimal fixation method. Some authors proposed fixation of the dorsoulnar fragment with a low-profile plate via additional 30 mm dorsal approach [1] or application of a single-fragment compression screw through a small incision [7]. In another work, the dorsoulnar fragment was targeted with volar plate screws after anatomical DRF reduction [53]. However, the screw length selection is under discussion because penetration of the dorsal cortex would lead to extensor tendon irritation or rupture, whereas missing screw support at the far cortex raises the question about insufficient stability of fixation.

In our study, no articular step-off of the lunate facet was observed irrespective of the fragmentation pattern.

The fracture model in Group 1 was the same as in previous work comparing the biomechanical competency of volar and double plated constructs representing AO/OTA 23-C2.1 fractures [24]. According to those findings, the two fixation methods revealed comparable biomechanical characteristics, which is in line with our study results.

In the present investigation, the magnitude of angular displacement of the lunate facet fragments with respect to the radial shaft after volar plating was related to the degree of comminution within the dorsal third of the lunate facet. In presence of such a comminution, considerably decreased angular displacement was observed following double plating. Due to the dorsal metaphyseal defect as a whole, simulated via a dorsal wedge-shaped osteotomy gap and being the principal source of instability, the tested specimens demonstrated only extraarticular displacement.

This study has some limitations inherent to all biomechanical investigations. A limited sample size of synthetic bones was used, resulting in restriction of the translation to generalized clinical applications. In agreement with previous work, the radii were consistently selected to be similar in size in order to provide less variation between the study groups [48]. An osteoporotic bone model was not explicitly tested. Despite this, we were able to detect several significant differences related to the different simulated fracture patterns, plate fixations and tested specimens’ positions. The fracture creation via osteotomizing did not necessarily obey the physical laws of real fracture mechanisms, however, it was performed because of standardization purposes. Moreover, fragmentation patterns of the lunate facet in real life can rarely be assigned to standardized groups with four or five fracture fragments. In addition, the anatomic reduction of articular fragments in controlled laboratory settings were only vaguely similar to the more complicated in-vivo situations. The setup for testing of the specimens in neutral position, flexion and extension was a gross simplification of the real fractured-bone situation and physiological loading conditions, not necessarily replicating the more complex in-vivo situations that may include torsional and bending moments. Furthermore, the biomechanics of in-vivo fracture healing could not be simulated. Instead, a direct post-operative primary stability situation without bone consolidation as worse-case scenario was explored.

## Conclusion

From a biomechanical perspective, fracture patterns featuring comminution of the lunate facet within its dorsal third significantly affect stability when comparing volar and double plate fixations of complex intraarticular distal radius fractures.

Double plating demonstrates superior fixation stability versus single volar plating only in case of lunate facet comminution within its dorsal third. In contrast, volar locked plating could achieve comparable stability versus double plate fixation when the dorsal third of the lunate facet is not separated by the fracture pattern. Both fixation methods indicate a potential of achieving absolute stability between the articular fragments.

From a clinical perspective, fracture fixation should be performed with a single volar plate only in cases with appropriate dorsoulnar fragment size of at least 6 mm when adequate anatomical reduction is possible.

## Data Availability

Access to the data will be provided upon request.

## Abbreviations

AD: Angular displacement
ANOVA: One-Way Analysis of Variance
AO: Arbeitsgemeinschaft für Osteosynthesefragen
AP: Anteroposterior
D: Double
DRF: Distal radius fracture
E: Extension
F: Flexion
GR: Group
ID: Interfragmentary displacement
K-wire: Kirschner wire
LCP: Locking Compression Plate
ML: Mediolateral
N: Neutral
OTA: Orthopaedic Trauma Association
P: *P*-value
PL: Plating
PMMA: Polymethylmethacrylate
ST: Stiffness
V: Volar
